# Can we enhance amphibians’ habitat restoration in the post-mining areas?

**DOI:** 10.1007/s11356-015-5279-8

**Published:** 2015-09-02

**Authors:** Krzysztof Klimaszewski, Ewa Pacholik, Adam Snopek

**Affiliations:** 1 Department of Animal Environment Biology, Animal Sciences Faculty, Warsaw University of Life Sciences—SGGW, Ciszewskiego st. 8, 02-786 Warsaw, Poland; 2 Department of Geoecology, Faculty of Geography and Regional Studies, University of Warsaw, Krakowskie Przedmieście 30, 00-927 Warsaw, Poland

**Keywords:** Post-mining terrains, Gravel pit, Nature restoration, Reclamation practices, Amphibians, Habitat quality indexes

## Abstract

The study was aimed to evaluate the selected improvements of nature restoration in a depleted gravel pit. The study site consisted of four water reservoirs of different shapes and sizes, flooded after the gravel extraction ended. Ecological succession monitoring, conducted by the Warsaw University of Life Sciences students associated in the Student Scientific Association of Animal Sciences Faculty since the completion of mining, have focused on amphibians. A twofold approach upheld amphibian species population dynamics, as well as selected habitat elements. The restoration practices dedicated to habitat conditions enhancing have been proved to be definitely effective and useful for similar sites.

## Introduction

Although the areas under intensive mining activity are characterized by severe environment degradation, some changes caused by the human activity may lead to formation of new ecosystems strictly dependent on that activity and in some respects exceeding the value of previous ecosystems (Kasprzyk 2009; Kasztelewicz 2010; Parusel and Karkosz 2012). Restoration of post-mining sites may significantly contribute to local biodiversity (Prach et al. 2011) and play an important role in protecting endangered animals and plant species and communities (Benes et al. 2003; Tropek et al. 2010). This process can be also used for recreational and educational purposes (Głogowska 2005; Wosik 2014).

Measures of ecosystem rehabilitation success can vary and may depend on the approach. Those measures should meet needs of different parties: community, government, industry, etc. (Bell 2001). From an ecological point of view, the success of restoration programs may be investigated by plant species diversity, density and rate of their succession (Pietrzykowski 2008; Prach et al. 2013; Rehounkova and Prach 2006, 2008), nutrient cycling and soil development (Pietrzykowski and Krzaklewski 2007), animal species colonization, and increase of their habitat suitability (Gould and Mackey 2015).

Majority of abandoned surface-mined terrains in Central Europe are renovated not only into forest habitats (Korjus et al. 2014; Pietrzykowski 2008) but also into aquatic ecosystems with water reservoirs created as a result of post-mining terrains reclamation (Kasprzak and Raszka 2009). They may become a suitable habitat for rare animal species connected with wetlands, enhancing their regional habitat conditions (Kasztelewicz 2010).

**Fig. 1:**
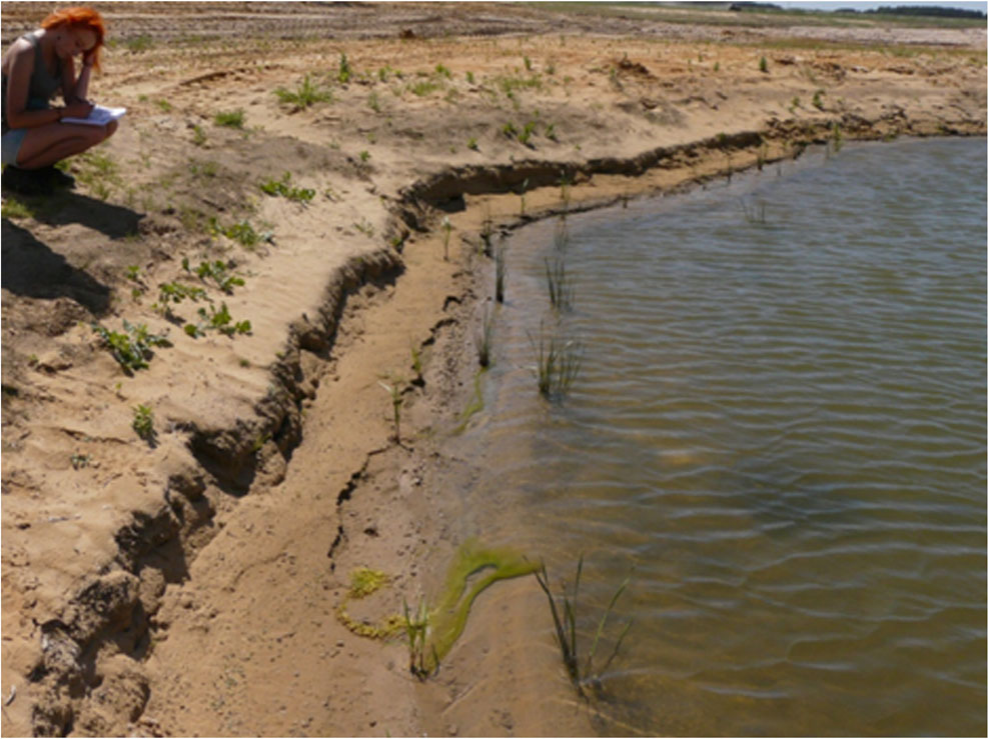
Water reservoir 3A with newly planted reed bed, May 2012 (Photo: A. Snopek)

This issue absorbed the Department of Environmental Protection of the CEMEX Polska company—one of the leading crushed stone producers in Poland (Kabziński 2010), owning the Sitno gravel pit flooded after exploitation in 2008–2011, located on the meadow terrace of the Narew River (Sitno, Maków County, Masovian Voivodeship, Central Poland)—aiming at not only protecting but also enhancing local biodiversity in mining sites. The traditional reclamation project has been extended by the experts from Warsaw Society for Protection of Birds in order to improve habitat conditions for selected animal groups and minimize negative impact on surrounding habitats (Poławski et al. 2011). Recommendations contained shoreline and slope formation (including shallowing of the water reservoirs and flattening of their banks), as well as planting the shores and surroundings with native plant species typical for adequate natural and seminatural habitats in the region—mainly with the grey willow *Salix cinerea*, the reed mannagrass *Glyceria maxima* and the lesser bulrush *Typha angustifolia* (Fig. 1), with a small admixture of the common rush *Juncus effusus*. Although the terrestrial part of the site has been recommended to be sown mainly with the sheep fescue *Festuca ovina* and the red fescue *Festuca rubra* according to Rogalski and Prajs (2006), the banks outside the reed beds have been recommended to be sown the creeping bentgrass *Agrostis stolonifera*. These modifications were dedicated mainly to amphibians. This environmentally fragile group of animals (influenced by both aquatic and terrestrial habitats, as well as being a predator on a high level of ecological food chain) is a sensitive bioindicator of habitat changes.

## Methods

**Fig. 2:**
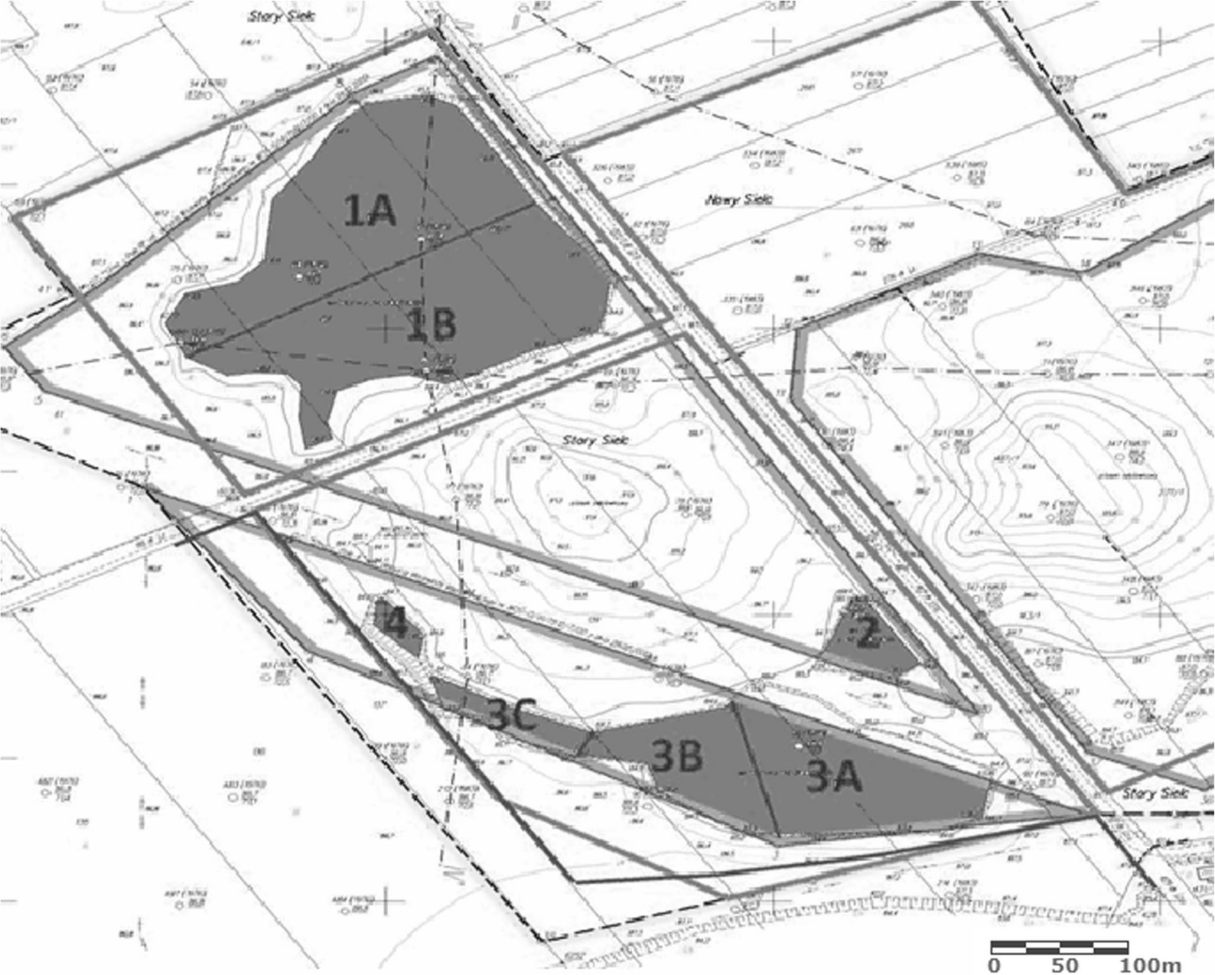
The study area on a map created using QGIS 2.6.1 (Pacholik 2014, modified)

Members of the Student Scientific Association of Animal Sciences Faculty (Warsaw University of Life Sciences) undertook the ecological succession monitoring focused on amphibians. Inventory using a combination of observation and vocal recognition focused on mating individuals (with occurrence of eggs and larvae as evidence) was conducted in 2012, 2013, and 2014, with 10 field controls since April to November each year. The study area divided into seven pieces—1A, 1B, 2, 3A, 3B, 3C, and 4—according to water reservoir depth, shoreline shape, and vegetation (Fig. 2) have been assessed according to its suitability for selected amphibian species by use of habitat quality indexes (Makomaska-Juchniewicz and Baran 2012) based on habitat suitability index (HSI) constructed for the great crested newt (Oldham et al. 2000). The results of this assessment, based on the conditions of 2013, have been compared to the results of the inventory of that year.

## Results

During the course of monitoring, 12 species of amphibians were registered: the European fire-bellied toad *Bombina bombina*, the common toad *Bufo bufo*, the natterjack toad *Epidalea calamita*, the European tree frog *Hyla arborea*, the smooth newt *Lissotriton vulgaris*, the common spadefoot *Pelobates fuscus*, the edible frog *Pelophylax esculentus*, the pool frog *Pelophylax lessonae*, the marsh frog *Pelophylax ridibundus*, the European green toad *Pseudepidalea viridis*, the moor frog *Rana arvalis*, and the common frog *Rana temporaria*. Table 1 contains the occurrence of the species selected as having habitat quality indexes, in particular, water reservoirs in 2013. Table 2 contains the habitat validation for those species.

**Table 1:** Occurrence of selected amphibian species in particular water reservoirs

	1A	1B	2	3A	3B	3C	4
*Pelobates fuscus*	0	0	0	0	1	1	0
*Bombina bombina*	0	0	1	0	0	1	1
*Epidalea calamita*	1	0	1	1	1	1	1
*Pseudepidalea viridis*	1	1	1	1	1	1	1
*Hyla arborea*	0	0	1	0	0	1	1
*Pelophylax lessonae*	2	2	2	2	2	2	2
*Rana arvalis*	1	0	0	0	1	1	1
*Pelophylax ridibundus*	2	2	2	2	2	2	2
*Rana temporaria*	2	1	2	2	2	2	2
*Pelophylax esculentus*	2	2	2	2	2	2	2

0, lack of individuals; 1, 1–10 individuals; 2, over 100 individuals (Pacholik and Klimaszewski 2013, modified)

**Table 2:** Habitat validation based on habitat quality index values

	1A	1B	2	3A	3B	3C	4
*Pelobates fuscus*	U1	U1	FV	U1	U1	FV	FV
*Bombina bombina*	U2	U2	FV	U1	U1	FV	FV
*Epidalea calamita*	FV	FV	FV	U1	FV	FV	FV
*Pseudepidalea viridis*	U1	FV	FV	FV	FV	FV	FV
*Hyla arborea*	U1	U2	FV	FV	FV	FV	FV
*Pelophylax lessonae*	FV	FV	FV	FV	FV	FV	FV
*Rana arvalis*	U1	U2	FV	FV	FV	FV	FV
*Pelophylax ridibundus*	U1	U1	U1	U1	U1	U1	U1
*Rana temporaria*	FV	FV	FV	FV	FV	FV	FV
*Pelophylax esculentus*	FV	U1	FV	FV	FV	FV	FV

FV—proper condition, U1—unsatisfactory condition, U2—bad condition

## Discussion

**Fig. 3:**
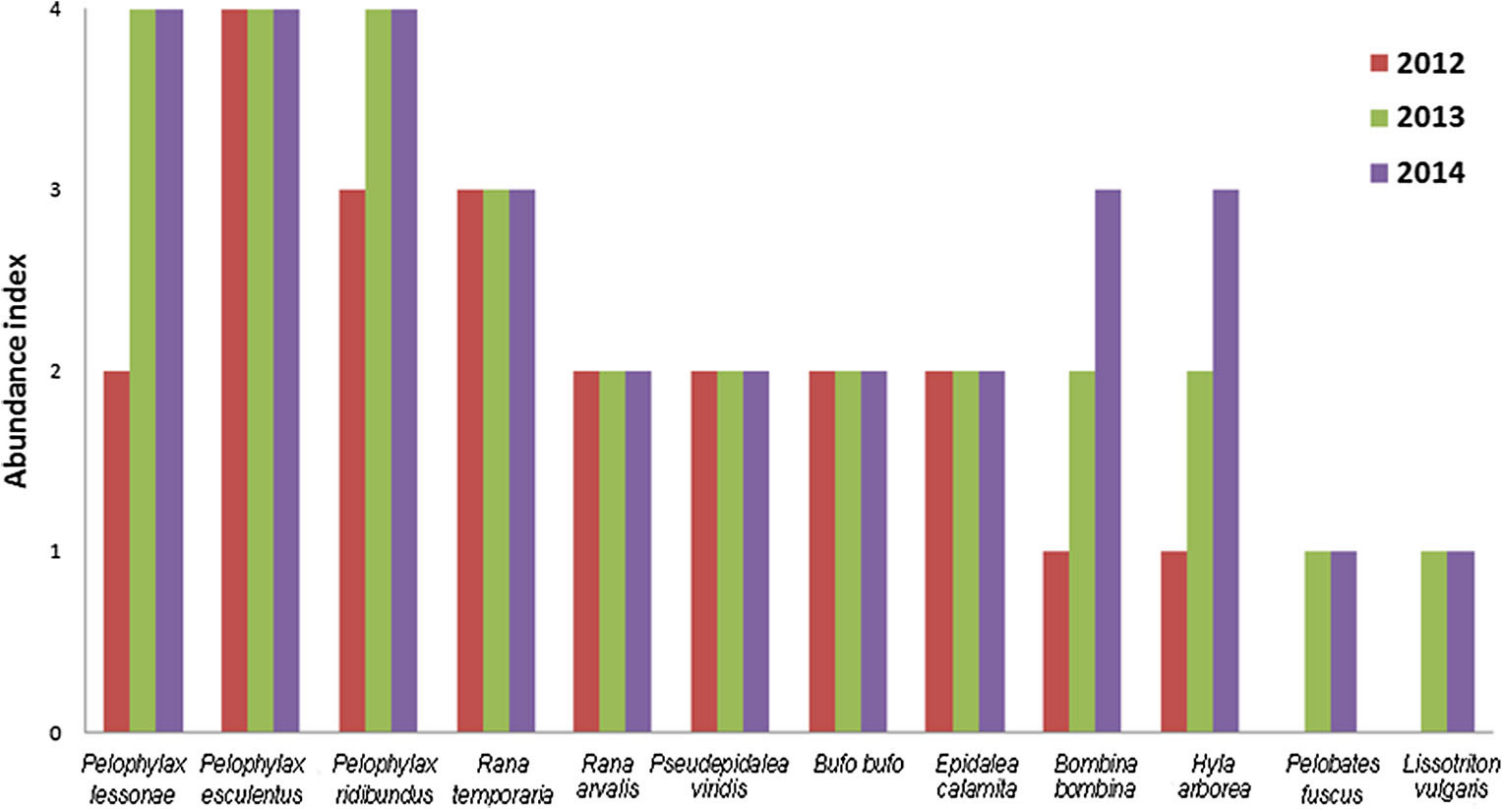
Occurrence of amphibian species in former Sitno gravel pit during three seasons; 0, lack of individuals; 1, 1–10 individuals; 2, 10–100 individuals; 3, 100–300 individuals; 4, over 300 individuals

The explosive amphibian colonization and breeding success was observed. Almost the whole range of amphibian species known from central Poland was found. The only species not found—the great crested newt—has requirements of more stable and forward stage of succession habitats (Edgar and Bird 2006). Autumnal observations (Pacholik 2014) revealed the dispersion of juveniles, available though favorable surroundings of the study area. The increase of the European fire-bellied toad and the European tree frog populations observed in 2014 (Fig. 3) proves the high value of newly formed habitats.

**Fig. 4:**
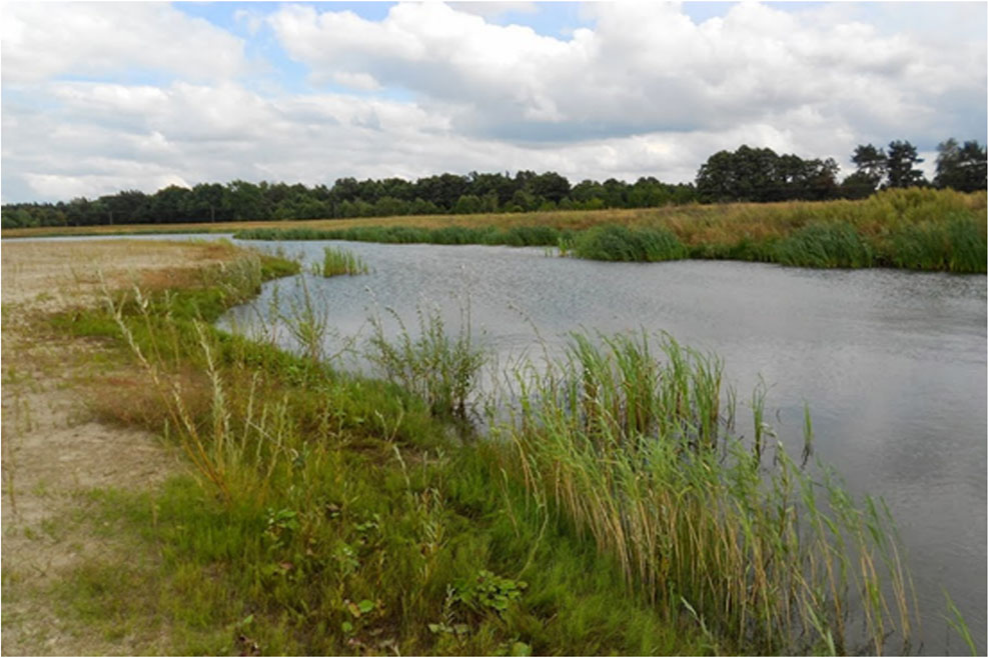
Water reservoir 3C (photo: E. Pacholik)

**Fig. 5:**
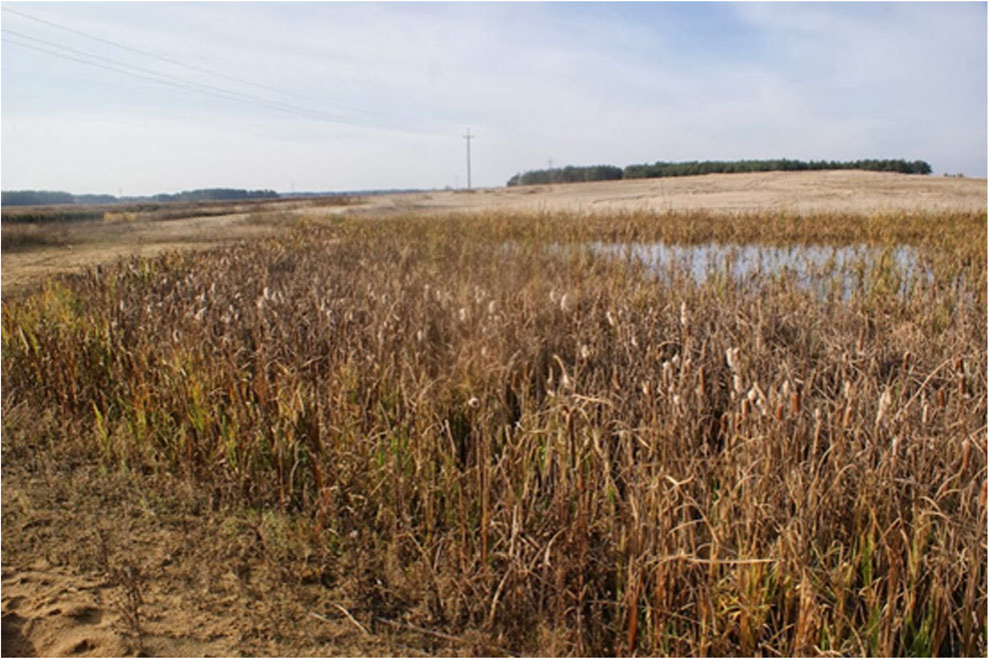
Water reservoir 2 (Photo: E. Pacholik)

**Fig. 6:**
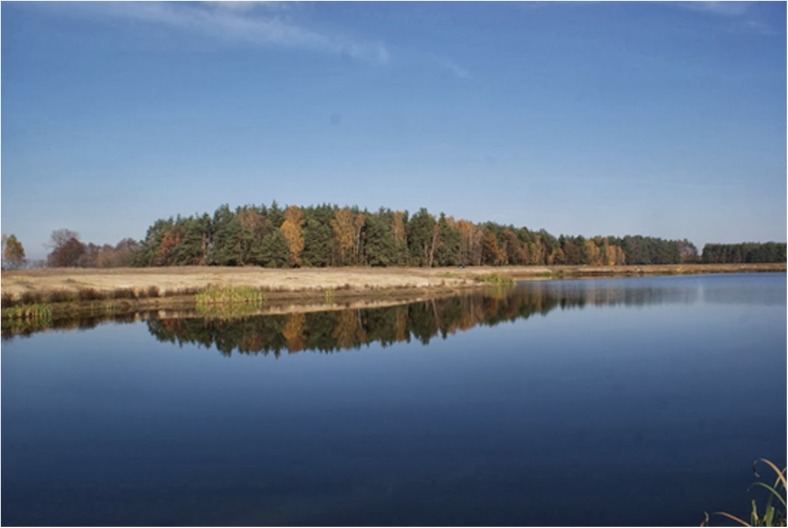
Water reservoir 1A (photo: E. Pacholik)

Water reservoirs 2, 3C (Fig. 4), and 4 proved to be the most convenient habitats for amphibians’ existence and breeding on account of small surface, large part of shallownesses, dense plant cover, and gentle shore slopes. Reservoir 2 was preferred by many species (especially *Pelophylax* sp.) but convenient conditions may not last in cause of the shallowness allowing overgrow completely (Fig. 5). Reservoir 1 (1A, 1B), with the highest shores slightly covered with plants, proved to be the least convenient for amphibians (Fig. 6).

The results of recent HSI-based research on the great crested newt from the same region (Klimaszewski and Białaś 2013) allowed expecting compatibility between the habitat quality index values and the occurrence of amphibian species, especially the most vulnerable ones, in the newly formed habitats. The particular species observations are fundamentally consistent with the adequate index values, especially for the pool frog and the European fire-bellied toad. Habitats convenient for the European fire-bellied toad—a species vulnerable to the most factors—proved to be most convenient for other species.

The difference between the plant covers of the study area and the other restored gravel pit in that close neighborhood, observed during the last grooving season, testifies to the effectiveness of the reclamation project extensions; without them, the grass succession would be slow and the shoreline vegetation, completely dominated by *Typha* sp. and the common reed *Phragmites australis*, would be less convenient for the registered amphibians preferring medium height and density of the reed beds (Makomaska-Juchniewicz and Baran 2012).

The applicable advantages of the Sitno case study in the near future refer to the forecasts of natural crushed stone production increase (Kozioł and Czaja 2010) and the participation of Mazovian Voivodeship in that production (Kasztelewicz 2010). The acceleration of exploitation and necessity of urgent reclamation undertaking in active mining sites as a result of that trend (Kasztelewicz 2010), as well as the expected collision of mining development with Natura 2000 sites in Poland (Kabziński 2010), decide on the importance of the issue. Methods developed in the Sitno site may be widely implemented in similar locations.

## Conclusions

- Flooded former gravel pits are potentially valuable habitats for amphibians
- Reclamation practices as shoreline and slope formation, as well as planting native plant species in the Sitno case were definitely effective
- Creating a large number of small water reservoirs of varied shape and depth with flattened shore slopes, with a developed shoreline, a large part of shallowness, and dense reed beds might become a best practice in the reclamation of gravel pits enhancing development of amphibian populations,
- European fire-bellied toad proves to be a good umbrella species for amphibians in ecological restoration of post-mining sites in Poland.
